# *tert*-Butyl 3-[2,2-bis­(ethoxy­carbon­yl)­vinyl]-2-methyl-1*H*-indole-1-carboxyl­ate

**DOI:** 10.1107/S1600536809009635

**Published:** 2009-03-25

**Authors:** M. Thenmozhi, T. Kavitha, V. Dhayalan, A. K. Mohanakrishnan, M. N. Ponnuswamy

**Affiliations:** a Centre of Advanced Study in Crystallography and Biophysics, University of Madras, Guindy Campus, Chennai 600 025, India; b Department of Organic Chemistry, University of Madras, Guindy Campus, Chennai 600 025, India

## Abstract

In the title compound, C_22_H_27_NO_6_, the indole ring system is planar and the ethoxy­carbonyl chains adopt extended conformations.  In the crystal, inversion dimers linked by pairs of C—H⋯O hydrogen bonds occur, resulting in *R*_2_^2^(16) dimers, which are inter­linked into a chain propagating along the *a* axis by π–π stacking inter­actions [centroid–centroid distance 3.5916 (9) Å].

## Related literature

For general background, see: Hood *et al.* (1992 ▶); Cram *et al.* (2001 ▶). For hybridization, see: Beddoes *et al.* (1986 ▶). For hydrogen-bond motifs, see: Bernstein *et al.* (1995 ▶).
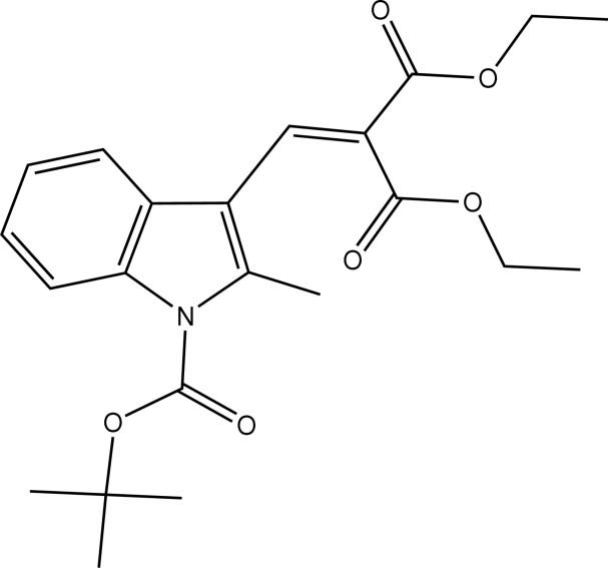


## Experimental

### 

#### Crystal data

C_22_H_27_NO_6_
*M* *_r_* = 401.45
Monoclinic, 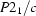

*a* = 9.1933 (3) Å
*b* = 21.8495 (6) Å
*c* = 10.7676 (3) Å
β = 96.510 (2)°
*V* = 2148.93 (11) Å^3^
*Z* = 4
Mo *K*α radiation
μ = 0.09 mm^−1^
*T* = 293 K
0.25 × 0.20 × 0.20 mm

#### Data collection

Bruker Kappa APEXII area-detector diffractometer
Absorption correction: multi-scan (*SADABS*; Sheldrick, 2001 ▶) *T*_min_ = 0.978, *T*_max_ = 0.982
24241 measured reflections
4766 independent reflections
3534 reflections with *I* > 2σ(*I*)
*R*_int_ = 0.030

#### Refinement

*R*[*F* ^2^ > 2σ(*F* ^2^)] = 0.049
*wR*(*F* ^2^) = 0.134
*S* = 1.04
4766 reflections
286 parameters
28 restraints
H-atom parameters constrained
Δρ_max_ = 0.25 e Å^−3^
Δρ_min_ = −0.28 e Å^−3^

### 

Data collection: *APEX2* (Bruker, 2004 ▶); cell refinement: *SAINT* (Bruker, 2004 ▶); data reduction: *SAINT*; program(s) used to solve structure: *SHELXS97* (Sheldrick, 2008 ▶); program(s) used to refine structure: *SHELXL97* (Sheldrick, 2008 ▶); molecular graphics: *ORTEP-3* (Farrugia, 1997 ▶); software used to prepare material for publication: *SHELXL97* and *PLATON* (Spek, 2009 ▶).

## Supplementary Material

Crystal structure: contains datablocks global, I. DOI: 10.1107/S1600536809009635/ci2766sup1.cif

Structure factors: contains datablocks I. DOI: 10.1107/S1600536809009635/ci2766Isup2.hkl

Additional supplementary materials:  crystallographic information; 3D view; checkCIF report

## Figures and Tables

**Table 1:** Hydrogen-bond geometry (Å, °)

*D*—H⋯*A*	*D*—H	H⋯*A*	*D*⋯*A*	*D*—H⋯*A*
C23—H23*C*⋯O6^i^	0.96	2.55	3.503 (3)	171

Symmetry code: (i) 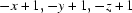.

**Fig. 1.**
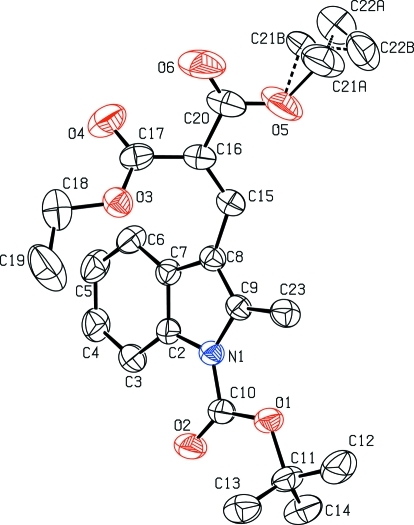
The molecular structure of the title compound, showing 30% probability displacement ellipsoids. H atoms have been omitted for clarity. Both disorder components are shown.

**Fig. 2.**
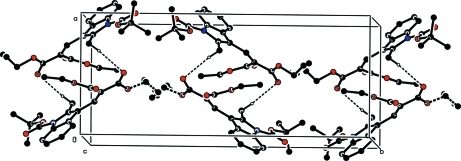
The packing of the molecules viewed down c axis, showing C—H···O interactions (dashed lines).

## supplementary crystallographic information

### Comment

Indole-carboxylate derivatives are known to be sensitive to *N*-methyl-D-aspartate (NMDA) antagonists and capable of reducing the damage associated with an ischemic insult in Mongolian gerbil hippocampal neurons (Hood *et al.*, 1992). Also the naturally occurring indole compounds can induce cell cycle to arrest the human breast cancer cells (Cram *et al.*, 2001).

The indole ring system is planar and both ethoxycarbonyl groups adopt extended conformation as can be seen from the torsion angles C17—O3—C18—C19 [169.7 (2)°], C18—O3—C17—C16 [-177.6 (2)°], C21A/C21B—O5—C20—C16 [-165.5 (4)° /170.9 (5)°] and C20—O5—C21A/C21B—C22A/C22B [-105.2 (6)° / -176.8 (5)°]. The sum of bond angles around N1 [359.96°] indicates that atom N1 exhibits *sp*^2^ hybridization (Beddoes *et al.*, 1986). In the *tert*-butoxycarbonyl group, the three C—C bond lengths lie between 1.501 (3) Å and 1.507 (3) Å, while the three *tert*-butyl C—C—C angles are in the range 110.6 (2)°–113.1 (2)°, indicating a slight opening up from the ideal tetrahedral value.

In the crystal structure, the molecules form *R*_2_^2^(16) dimers through paired C23—H23C···O6 hydrogen bonds (Bernstein *et al.*, 1995). A π-π stacking interaction is observed between pyrrole rings of moloecules at (*x*, *y*, *z*) and (2 - *x*, -*y*, -*z*), with a centroid to centroid distance of 3.5916 (9) Å.

### Experimental

To a solution of *tert*-butyl 3-formyl-2-methyl-1*H*-indole-1-carboxylate (4 g, 15.44 mmol) in dry benzene (120 ml), diethylmalonate (2.8 ml, 18.53 mmol), piperidine (6 drops) and acetic acid (3 drops) were added and refluxed in Dean-Stark apparatus for 48 h. Removal of solvent followed by recrystallization from methanol afforded the product as brown crystals.

### Refinement

The ethyl C atoms of the ethoxycarbonyl group are disordered over two positions (C21A/C22A and C21B/C22B) with refined occupancies of 0.58 (1) and 0.42 (1). The corresponding O—C and C—C bond distances involving the disordered atoms were restrained to 1.45 (1) Å and 1.53 (1) Å, respectively, and also their U^ij^ parameters were restrained to an approximate isotropic behaviour. H atoms were positioned geometrically (C—H = 0.93–0.97 Å) and allowed to ride on their parent atoms, with *U*_iso_(H) = 1.2–1.5(methyl)*U*_eq_(C).

### Crystal data

**Table:**

C_22_H_27_NO_6_	*F*(000) = 856
*M**_r_* = 401.45	*D*_x_ = 1.241 Mg m^−^^3^
Monoclinic, *P*2_1_/*c*	Mo *K*α radiation, λ = 0.71073 Å
Hall symbol: -P 2ybc	Cell parameters from 4766 reflections
*a* = 9.1933 (3) Å	θ = 2.1–27.2°
*b* = 21.8495 (6) Å	µ = 0.09 mm^−^^1^
*c* = 10.7676 (3) Å	*T* = 293 K
β = 96.510 (2)°	Block, brown
*V* = 2148.93 (11) Å^3^	0.25 × 0.20 × 0.20 mm
*Z* = 4	

### Data collection

**Table:**

Bruker Kappa APEXII area-detector diffractometer	4766 independent reflections
Radiation source: fine-focus sealed tube	3534 reflections with *I* > 2σ(*I*)
graphite	*R*_int_ = 0.030
ω and φ scans	θ_max_ = 27.2°, θ_min_ = 2.1°
Absorption correction: multi-scan (*SADABS*; Sheldrick, 2001)	*h* = −11→11
*T*_min_ = 0.978, *T*_max_ = 0.982	*k* = −27→28
24241 measured reflections	*l* = −13→13

### Refinement

**Table:**

Refinement on *F*^2^	Primary atom site location: structure-invariant direct methods
Least-squares matrix: full	Secondary atom site location: difference Fourier map
*R*[*F*^2^ > 2σ(*F*^2^)] = 0.049	Hydrogen site location: inferred from neighbouring sites
*wR*(*F*^2^) = 0.134	H-atom parameters constrained
*S* = 1.04	*w* = 1/[σ^2^(*F*_o_^2^) + (0.0561*P*)^2^ + 0.6467*P*] where *P* = (*F*_o_^2^ + 2*F*_c_^2^)/3
4766 reflections	(Δ/σ)_max_ = 0.001
286 parameters	Δρ_max_ = 0.25 e Å^−^^3^
28 restraints	Δρ_min_ = −0.28 e Å^−^^3^

### Special details

**Table:**

Geometry. All e.s.d.'s (except the e.s.d. in the dihedral angle between two l.s. planes) are estimated using the full covariance matrix. The cell e.s.d.'s are taken into account individually in the estimation of e.s.d.'s in distances, angles and torsion angles; correlations between e.s.d.'s in cell parameters are only used when they are defined by crystal symmetry. An approximate (isotropic) treatment of cell e.s.d.'s is used for estimating e.s.d.'s involving l.s. planes.
Refinement. Refinement of *F*^2^ against ALL reflections. The weighted *R*-factor *wR* and goodness of fit *S* are based on *F*^2^, conventional *R*-factors *R* are based on *F*, with *F* set to zero for negative *F*^2^. The threshold expression of *F*^2^ > σ(*F*^2^) is used only for calculating *R*-factors(gt) *etc*. and is not relevant to the choice of reflections for refinement. *R*-factors based on *F*^2^ are statistically about twice as large as those based on *F*, and *R*- factors based on ALL data will be even larger.

### Fractional atomic coordinates and isotropic or equivalent isotropic displacement parameters (Å^2^)

**Table:**

	*x*	*y*	*z*	*U*_iso_*/*U*_eq_	Occ. (<1)
O1	0.11345 (14)	0.63504 (5)	0.68637 (11)	0.0507 (3)	
O2	0.00198 (17)	0.68868 (6)	0.52523 (12)	0.0639 (4)	
O3	0.39872 (13)	0.51263 (5)	0.24128 (12)	0.0508 (3)	
O4	0.34111 (19)	0.43222 (8)	0.11765 (15)	0.0797 (5)	
O5	0.3720 (2)	0.33528 (7)	0.4564 (2)	0.0942 (6)	
O6	0.49107 (19)	0.34337 (7)	0.2910 (2)	0.0947 (6)	
N1	0.09171 (14)	0.59345 (5)	0.49600 (12)	0.0370 (3)	
C2	0.03282 (17)	0.58731 (7)	0.37025 (14)	0.0378 (3)	
C3	−0.06844 (19)	0.62313 (8)	0.29745 (16)	0.0482 (4)	
H3	−0.1061	0.6587	0.3287	0.058*	
C4	−0.1107 (2)	0.60389 (9)	0.17750 (17)	0.0572 (5)	
H4	−0.1775	0.6273	0.1263	0.069*	
C5	−0.0566 (2)	0.55057 (10)	0.13070 (16)	0.0571 (5)	
H5	−0.0877	0.5387	0.0490	0.069*	
C6	0.0423 (2)	0.51497 (9)	0.20315 (16)	0.0491 (4)	
H6	0.0776	0.4790	0.1715	0.059*	
C7	0.08915 (17)	0.53353 (7)	0.32463 (14)	0.0386 (3)	
C8	0.18327 (17)	0.50633 (7)	0.42574 (15)	0.0381 (3)	
C9	0.18175 (16)	0.54260 (7)	0.52819 (14)	0.0368 (3)	
C10	0.06355 (18)	0.64434 (7)	0.56895 (15)	0.0425 (4)	
C11	0.1044 (2)	0.68359 (8)	0.78201 (17)	0.0514 (4)	
C12	0.1872 (3)	0.65426 (12)	0.89548 (19)	0.0792 (7)	
H12A	0.1399	0.6167	0.9140	0.119*	
H12B	0.1885	0.6815	0.9655	0.119*	
H12C	0.2857	0.6459	0.8792	0.119*	
C13	0.1810 (2)	0.74056 (9)	0.7464 (2)	0.0657 (5)	
H13A	0.2768	0.7302	0.7256	0.099*	
H13B	0.1897	0.7687	0.8153	0.099*	
H13C	0.1256	0.7592	0.6754	0.099*	
C14	−0.0535 (2)	0.69411 (10)	0.7993 (2)	0.0672 (6)	
H14A	−0.1032	0.7120	0.7248	0.101*	
H14B	−0.0595	0.7213	0.8686	0.101*	
H14C	−0.0986	0.6558	0.8157	0.101*	
C15	0.26345 (17)	0.44895 (7)	0.42693 (16)	0.0431 (4)	
H15	0.2655	0.4263	0.5002	0.052*	
C16	0.33461 (18)	0.42406 (7)	0.33802 (17)	0.0465 (4)	
C17	0.35749 (19)	0.45525 (8)	0.21940 (18)	0.0513 (4)	
C18	0.4202 (3)	0.55038 (11)	0.1348 (2)	0.0752 (6)	
H18A	0.3363	0.5474	0.0718	0.090*	
H18B	0.5067	0.5373	0.0981	0.090*	
C19	0.4382 (4)	0.61385 (13)	0.1811 (3)	0.1133 (11)	
H19A	0.3498	0.6270	0.2128	0.170*	
H19B	0.4585	0.6402	0.1139	0.170*	
H19C	0.5179	0.6156	0.2468	0.170*	
C20	0.4073 (2)	0.36390 (8)	0.3563 (2)	0.0626 (5)	
C21A	0.4535 (8)	0.2800 (2)	0.5168 (11)	0.093 (2)	0.580 (10)
H21A	0.5473	0.2741	0.4853	0.111*	0.580 (10)
H21B	0.4684	0.2836	0.6071	0.111*	0.580 (10)
C22A	0.3500 (8)	0.2300 (3)	0.4765 (13)	0.089 (3)	0.420 (10)
H22A	0.3897	0.1917	0.5081	0.134*	0.420 (10)
H22B	0.3350	0.2286	0.3869	0.134*	0.420 (10)
H22C	0.2582	0.2374	0.5084	0.134*	0.420 (10)
C21B	0.4319 (10)	0.2723 (3)	0.4519 (9)	0.0645 (19)	0.420 (10)
H21C	0.3936	0.2511	0.3760	0.077*	0.420 (10)
H21D	0.5380	0.2724	0.4591	0.077*	0.420 (10)
C22B	0.3768 (8)	0.2453 (3)	0.5638 (9)	0.096 (2)	0.580 (10)
H22D	0.4068	0.2033	0.5719	0.144*	0.580 (10)
H22E	0.2718	0.2475	0.5552	0.144*	0.580 (10)
H22F	0.4160	0.2675	0.6369	0.144*	0.580 (10)
C23	0.26713 (19)	0.53322 (8)	0.65178 (16)	0.0478 (4)	
H23A	0.3283	0.4978	0.6487	0.072*	
H23B	0.2014	0.5273	0.7139	0.072*	
H23C	0.3271	0.5685	0.6729	0.072*	

### Atomic displacement parameters (Å^2^)

**Table:**

	*U*^11^	*U*^22^	*U*^33^	*U*^12^	*U*^13^	*U*^23^
O1	0.0686 (8)	0.0437 (6)	0.0384 (6)	0.0115 (6)	−0.0001 (6)	−0.0066 (5)
O2	0.0922 (10)	0.0455 (7)	0.0517 (8)	0.0258 (7)	−0.0018 (7)	−0.0023 (6)
O3	0.0479 (7)	0.0487 (7)	0.0557 (8)	0.0009 (5)	0.0059 (6)	0.0031 (5)
O4	0.0929 (12)	0.0852 (11)	0.0638 (9)	−0.0061 (9)	0.0205 (8)	−0.0259 (8)
O5	0.0943 (12)	0.0479 (8)	0.1461 (17)	0.0286 (8)	0.0386 (12)	0.0268 (10)
O6	0.0797 (11)	0.0628 (10)	0.1485 (17)	0.0245 (8)	0.0437 (11)	−0.0134 (10)
N1	0.0403 (7)	0.0349 (6)	0.0353 (7)	0.0041 (5)	0.0019 (5)	−0.0001 (5)
C2	0.0400 (8)	0.0386 (8)	0.0349 (8)	0.0015 (6)	0.0047 (6)	0.0018 (6)
C3	0.0525 (10)	0.0458 (9)	0.0454 (10)	0.0110 (7)	0.0022 (8)	0.0056 (7)
C4	0.0592 (11)	0.0679 (12)	0.0421 (10)	0.0134 (9)	−0.0045 (8)	0.0110 (8)
C5	0.0597 (11)	0.0756 (13)	0.0348 (9)	0.0058 (10)	−0.0003 (8)	−0.0023 (8)
C6	0.0504 (10)	0.0566 (10)	0.0406 (9)	0.0053 (8)	0.0058 (7)	−0.0074 (7)
C7	0.0378 (8)	0.0409 (8)	0.0375 (8)	0.0012 (6)	0.0058 (6)	0.0008 (6)
C8	0.0361 (8)	0.0367 (8)	0.0416 (9)	0.0019 (6)	0.0050 (6)	0.0006 (6)
C9	0.0337 (7)	0.0344 (7)	0.0419 (8)	0.0017 (6)	0.0027 (6)	0.0028 (6)
C10	0.0488 (9)	0.0369 (8)	0.0417 (9)	0.0052 (7)	0.0043 (7)	−0.0016 (7)
C11	0.0596 (11)	0.0500 (10)	0.0446 (10)	0.0031 (8)	0.0055 (8)	−0.0149 (7)
C12	0.1044 (18)	0.0845 (16)	0.0454 (11)	0.0108 (14)	−0.0056 (11)	−0.0163 (11)
C13	0.0655 (13)	0.0569 (11)	0.0758 (14)	−0.0066 (9)	0.0130 (11)	−0.0185 (10)
C14	0.0648 (13)	0.0679 (13)	0.0716 (14)	−0.0075 (10)	0.0198 (11)	−0.0251 (10)
C15	0.0403 (8)	0.0369 (8)	0.0517 (10)	0.0015 (6)	0.0037 (7)	0.0014 (7)
C16	0.0400 (9)	0.0372 (8)	0.0625 (11)	0.0010 (7)	0.0070 (8)	−0.0074 (7)
C17	0.0416 (9)	0.0527 (10)	0.0606 (11)	0.0030 (8)	0.0098 (8)	−0.0116 (9)
C18	0.0777 (15)	0.0826 (16)	0.0658 (14)	−0.0049 (12)	0.0107 (11)	0.0210 (12)
C19	0.147 (3)	0.0703 (17)	0.117 (2)	−0.0217 (17)	−0.009 (2)	0.0326 (16)
C20	0.0486 (10)	0.0378 (9)	0.1030 (17)	0.0015 (8)	0.0151 (11)	−0.0059 (10)
C21A	0.107 (4)	0.057 (3)	0.112 (5)	0.024 (3)	0.001 (4)	0.002 (4)
C22A	0.094 (4)	0.072 (4)	0.101 (6)	0.004 (3)	0.008 (4)	0.009 (4)
C21B	0.090 (4)	0.038 (3)	0.067 (4)	0.022 (3)	0.014 (3)	0.011 (3)
C22B	0.117 (4)	0.073 (3)	0.102 (5)	0.028 (3)	0.031 (4)	0.037 (3)
C23	0.0466 (9)	0.0463 (9)	0.0477 (10)	0.0074 (7)	−0.0065 (7)	0.0002 (7)

### Geometric parameters (Å, °)

**Table:**

O1—C10	1.311 (2)	C12—H12C	0.96
O1—C11	1.4874 (19)	C13—H13A	0.96
O2—C10	1.1917 (19)	C13—H13B	0.96
O3—C17	1.323 (2)	C13—H13C	0.96
O3—C18	1.444 (2)	C14—H14A	0.96
O4—C17	1.200 (2)	C14—H14B	0.96
O5—C20	1.318 (3)	C14—H14C	0.96
O5—C21B	1.484 (6)	C15—C16	1.335 (2)
O5—C21A	1.528 (6)	C15—H15	0.93
O6—C20	1.188 (2)	C16—C20	1.478 (2)
N1—C10	1.402 (2)	C16—C17	1.484 (3)
N1—C9	1.4053 (19)	C18—C19	1.477 (4)
N1—C2	1.4065 (19)	C18—H18A	0.97
C2—C3	1.389 (2)	C18—H18B	0.97
C2—C7	1.396 (2)	C19—H19A	0.96
C3—C4	1.372 (3)	C19—H19B	0.96
C3—H3	0.93	C19—H19C	0.96
C4—C5	1.384 (3)	C21A—C22A	1.481 (7)
C4—H4	0.93	C21A—H21A	0.97
C5—C6	1.371 (3)	C21A—H21B	0.97
C5—H5	0.93	C22A—H22A	0.96
C6—C7	1.390 (2)	C22A—H22B	0.96
C6—H6	0.93	C22A—H22C	0.96
C7—C8	1.440 (2)	C21B—C22B	1.482 (6)
C8—C9	1.360 (2)	C21B—H21C	0.97
C8—C15	1.454 (2)	C21B—H21D	0.97
C9—C23	1.481 (2)	C22B—H22D	0.96
C11—C13	1.501 (3)	C22B—H22E	0.96
C11—C14	1.502 (3)	C22B—H22F	0.96
C11—C12	1.507 (3)	C23—H23A	0.96
C12—H12A	0.96	C23—H23B	0.96
C12—H12B	0.96	C23—H23C	0.96
			
C10—O1—C11	121.09 (13)	H14A—C14—H14C	109.5
C17—O3—C18	117.53 (16)	H14B—C14—H14C	109.5
C20—O5—C21B	106.6 (3)	C16—C15—C8	129.37 (16)
C20—O5—C21A	124.6 (4)	C16—C15—H15	115.3
C10—N1—C9	129.03 (13)	C8—C15—H15	115.3
C10—N1—C2	122.65 (12)	C15—C16—C20	121.19 (18)
C9—N1—C2	108.28 (12)	C15—C16—C17	123.98 (15)
C3—C2—C7	121.85 (15)	C20—C16—C17	114.67 (16)
C3—C2—N1	130.54 (15)	O4—C17—O3	124.32 (19)
C7—C2—N1	107.54 (13)	O4—C17—C16	125.33 (18)
C4—C3—C2	117.33 (16)	O3—C17—C16	110.35 (15)
C4—C3—H3	121.3	O3—C18—C19	106.8 (2)
C2—C3—H3	121.3	O3—C18—H18A	110.4
C3—C4—C5	121.69 (17)	C19—C18—H18A	110.4
C3—C4—H4	119.2	O3—C18—H18B	110.4
C5—C4—H4	119.2	C19—C18—H18B	110.4
C6—C5—C4	120.87 (17)	H18A—C18—H18B	108.6
C6—C5—H5	119.6	C18—C19—H19A	109.5
C4—C5—H5	119.6	C18—C19—H19B	109.5
C5—C6—C7	118.98 (17)	H19A—C19—H19B	109.5
C5—C6—H6	120.5	C18—C19—H19C	109.5
C7—C6—H6	120.5	H19A—C19—H19C	109.5
C6—C7—C2	119.28 (15)	H19B—C19—H19C	109.5
C6—C7—C8	133.34 (15)	O6—C20—O5	122.79 (19)
C2—C7—C8	107.25 (13)	O6—C20—C16	124.8 (2)
C9—C8—C7	108.20 (13)	O5—C20—C16	112.41 (17)
C9—C8—C15	123.19 (14)	C22A—C21A—O5	101.0 (5)
C7—C8—C15	128.55 (15)	C22A—C21A—H21A	111.6
C8—C9—N1	108.70 (13)	O5—C21A—H21A	111.6
C8—C9—C23	126.54 (14)	C22A—C21A—H21B	111.6
N1—C9—C23	124.65 (14)	O5—C21A—H21B	111.6
O2—C10—O1	127.26 (15)	H21A—C21A—H21B	109.4
O2—C10—N1	122.20 (15)	C21A—C22A—H22A	109.5
O1—C10—N1	110.53 (13)	C21A—C22A—H22B	109.5
O1—C11—C13	110.46 (15)	H22A—C22A—H22B	109.5
O1—C11—C14	109.02 (15)	C21A—C22A—H22C	109.5
C13—C11—C14	113.14 (17)	H22A—C22A—H22C	109.5
O1—C11—C12	101.26 (14)	H22B—C22A—H22C	109.5
C13—C11—C12	110.58 (18)	C22B—C21B—O5	100.4 (4)
C14—C11—C12	111.74 (19)	C22B—C21B—H21C	111.7
C11—C12—H12A	109.5	O5—C21B—H21C	111.7
C11—C12—H12B	109.5	C22B—C21B—H21D	111.7
H12A—C12—H12B	109.5	O5—C21B—H21D	111.7
C11—C12—H12C	109.5	H21C—C21B—H21D	109.5
H12A—C12—H12C	109.5	C21B—C22B—H22D	109.5
H12B—C12—H12C	109.5	C21B—C22B—H22E	109.5
C11—C13—H13A	109.5	H22D—C22B—H22E	109.5
C11—C13—H13B	109.5	C21B—C22B—H22F	109.5
H13A—C13—H13B	109.5	H22D—C22B—H22F	109.5
C11—C13—H13C	109.5	H22E—C22B—H22F	109.5
H13A—C13—H13C	109.5	C9—C23—H23A	109.5
H13B—C13—H13C	109.5	C9—C23—H23B	109.5
C11—C14—H14A	109.5	H23A—C23—H23B	109.5
C11—C14—H14B	109.5	C9—C23—H23C	109.5
H14A—C14—H14B	109.5	H23A—C23—H23C	109.5
C11—C14—H14C	109.5	H23B—C23—H23C	109.5
			
C10—N1—C2—C3	−6.4 (3)	C2—N1—C10—O2	−8.8 (3)
C9—N1—C2—C3	175.54 (17)	C9—N1—C10—O1	−11.4 (2)
C10—N1—C2—C7	176.75 (14)	C2—N1—C10—O1	171.02 (14)
C9—N1—C2—C7	−1.29 (16)	C10—O1—C11—C13	−57.4 (2)
C7—C2—C3—C4	−0.5 (3)	C10—O1—C11—C14	67.5 (2)
N1—C2—C3—C4	−176.98 (17)	C10—O1—C11—C12	−174.57 (17)
C2—C3—C4—C5	0.8 (3)	C9—C8—C15—C16	−143.93 (18)
C3—C4—C5—C6	−0.2 (3)	C7—C8—C15—C16	39.3 (3)
C4—C5—C6—C7	−0.7 (3)	C8—C15—C16—C20	−178.54 (16)
C5—C6—C7—C2	0.9 (3)	C8—C15—C16—C17	6.3 (3)
C5—C6—C7—C8	176.11 (18)	C18—O3—C17—O4	2.3 (3)
C3—C2—C7—C6	−0.3 (2)	C18—O3—C17—C16	−177.62 (16)
N1—C2—C7—C6	176.85 (14)	C15—C16—C17—O4	−135.4 (2)
C3—C2—C7—C8	−176.65 (15)	C20—C16—C17—O4	49.2 (3)
N1—C2—C7—C8	0.51 (17)	C15—C16—C17—O3	44.5 (2)
C6—C7—C8—C9	−175.13 (18)	C20—C16—C17—O3	−130.88 (16)
C2—C7—C8—C9	0.47 (18)	C17—O3—C18—C19	169.7 (2)
C6—C7—C8—C15	2.0 (3)	C21B—O5—C20—O6	−10.6 (5)
C2—C7—C8—C15	177.64 (15)	C21A—O5—C20—O6	13.0 (5)
C7—C8—C9—N1	−1.27 (17)	C21B—O5—C20—C16	170.9 (5)
C15—C8—C9—N1	−178.63 (14)	C21A—O5—C20—C16	−165.5 (4)
C7—C8—C9—C23	−177.64 (15)	C15—C16—C20—O6	−167.6 (2)
C15—C8—C9—C23	5.0 (3)	C17—C16—C20—O6	8.0 (3)
C10—N1—C9—C8	−176.27 (15)	C15—C16—C20—O5	10.9 (3)
C2—N1—C9—C8	1.60 (17)	C17—C16—C20—O5	−173.58 (18)
C10—N1—C9—C23	0.2 (2)	C20—O5—C21A—C22A	−105.2 (6)
C2—N1—C9—C23	178.06 (15)	C21B—O5—C21A—C22A	−49.7 (9)
C11—O1—C10—O2	−3.5 (3)	C20—O5—C21B—C22B	−176.8 (5)
C11—O1—C10—N1	176.67 (14)	C21A—O5—C21B—C22B	48.2 (9)
C9—N1—C10—O2	168.77 (17)		

### Hydrogen-bond geometry (Å, °)

**Table:**

*D*—H···*A*	*D*—H	H···*A*	*D*···*A*	*D*—H···*A*
C23—H23C···O6^i^	0.96	2.55	3.503 (3)	171

Symmetry codes: (i) −*x*+1, −*y*+1, −*z*+1.
